# P-1038. Clinical Analysis of Nosocomial Candidemia: Species Profile, Resistance Patterns and Mortality Determinants

**DOI:** 10.1093/ofid/ofae631.1228

**Published:** 2025-01-29

**Authors:** Deepak Kumar, Monika Chaudhary, Naresh Kumar Midha, Durga Shankar Meena, Gopal Krishna Bohra, M K Garg, Nikhil Kothari, Pradeep Bhatia

**Affiliations:** All India Institute of Medical Sciences, Jodhpur (India), Jodhpur, Rajasthan, India; AIIMS, JODHPUR, JODHPUR, Rajasthan, India; AIIMS, Jodhpur, Rajasthan, India; AIIMS, Jodhpur, Rajasthan, India; AIIMS, Jodhpur, Rajasthan, India; AIIMS, Jodhpur, Rajasthan, India; AIIMS, JODHPUR, JODHPUR, Rajasthan, India; AIIMS, JODHPUR, JODHPUR, Rajasthan, India

## Abstract

**Background:**

Candidemia has become one of the commonest nosocomial blood stream infection (BSI) secondary to infected central venous catheters or surgical site infections. Prompt diagnosis followed by early treatment is needed to reduce the high mortality associated with it. Hence this study was conducted to analyse the epidemiology, clinical characteristics, species distribution, resistance profile, outcome and factors related to outcome in nosocomial candidemia.

**Figure:**
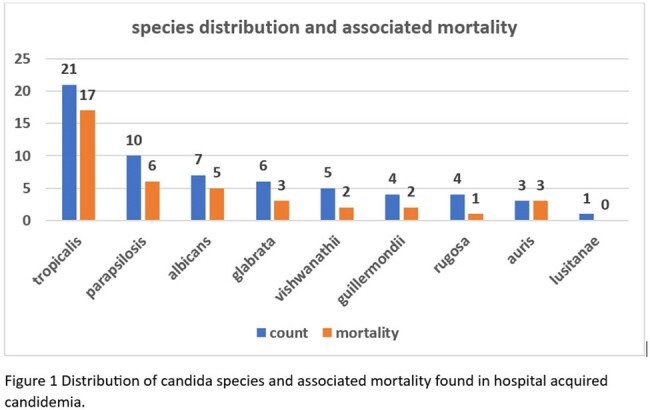

**Methods:**

It was a retrospective observational analysis done for the period of 1 year in a tertiary health care centre of western Rajasthan from 1^st^ July 2022 to 30^th^ June 2023. All adult patients admitted in multispeciality intensive care unit (ICU) for more than 48 hours and developing candidemia were included and those patients with a single blood fungal culture positive from the sample obtained from indwelling central venous catheter were excluded as colonisers. Data was collected by regular AMS rounds and was analysed using SPSS version 20.

**Figure:**
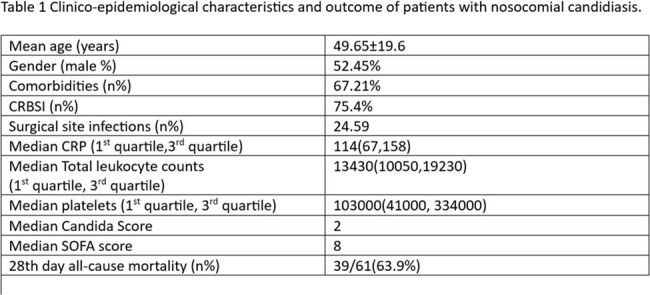

**Results:**

Out of total 76 cases of candidemia,61 were included in the study. As shown in figure 1, the most common species isolated was Candida tropicalis followed by Candida parapsilosis.both were associated with high mortality. Clinicoepidemiological characters are described in table1. Overall, all cause 28^th^ day mortality was 63.9 %. Highest resistance was found for the azoles. As depicted in table 2, on univariate and multivariate analysis infection with Candida tropicalis, use of inotropes, ventilator use, and high SOFA scores were the independent factors affecting mortality in candidemia.

**Figure:**
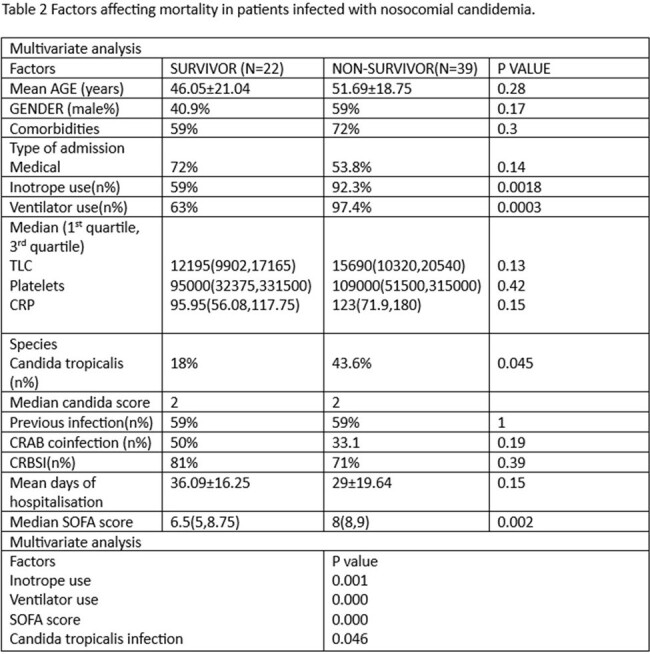

**Conclusion:**

Candidemia is a nosocomial infection with very high mortality which needs to be diagnosed early. Candida tropicalis being the most frequent species isolated in our ICU setup with a significantly high mortality.

**Disclosures:**

**All Authors**: No reported disclosures

